# 

**Published:** 1900-08-25

**Authors:** 


### NOTICE TO CORRESPONDENTS.

All MS., Letters, Books for Review, and other matters intended for the Editor should be addressed The Editor, "The Hospital," The Hospital Building, 28 & 29, Southampton Street, Strand, W.C.

The Editor cannot undertake to return rejected MS., even when accompanied by stamped directed envelope.

**Notice to Advertisers and Subscribers.**

All Advertisements, Orders for Copies of The Hospital, and Business Communications should be addressed to **THE MANAGER (Not to the Editor),** The Hospital, The Hospital Building, 28 & 29, Southampton Street, Strand, London, W.C.

The Cost of Subscription, post free, is as follows:—Per week, 2½d.; per quarter, 2s. 8d.; per half-year, 5s. 3d.; per annum, 10s. 6d. Foreign:—Per annum, 15s. 2d., payable, in all cases, in advance.

**NOTICE.—Vol. XXVII. of "The Hospital" (October, 1899, to April, 1900), in handsome cloth binding, is now on sale at the Office of this Journal, price 7s. 6d. Cases for binding the Numbers contained in this Volume 1s. 6d. Index to Vol. XXVII., 2½d., post free.**

**The Monthly Part for September will be ready on September 1st. Price 6d., by post 8d.**

### BOOKS RECEIVED.

John Wright and Co.

"Operative and Practical Surgery." By Thomas Carwardine, M.S. Lond., F.R.C.S.

Skeffington and Son.

"The Humours of a Hydro." By Dagney Major.

Baillière, Tindall, and Cox.

"Hernia: Its Etiology, Symptoms, and Treatment." By W. McAdam Eccles, M.S., F.R.C.S.

"The Principles and Practice of Hydrotherapy." By Simon Baruch, M.D.

"The Permanganate Treatment of Opium and Morphine Poisoning." By Dr. William Ovid Moor.

The Great Eastern Railway Company.

"Tourist Guide to the Continent." Edited by Percy Lindley.

W. B. Clive.

"First Stage Botany." By Alfred J. Ewart, D.Sc., Ph.D., F.L.S.

William Hodge and Co., Glasgow.

"Annual Reports to the County Council of Dumbarton and to the County Council of Stirling." By John C. McVail, M.D., D.P.H., F.R.S.E., Medical Officer of Health.

Charles J. Thynne.

"The Special Evils of the Streets of London." A Speech by Samuel Smith, Esq., M.P.

### Scraps and Gleanings.

The number of in-patients treated during the year at the Bridlington Cottage Hospital was 221, against 177 last year.

The Worshipful Company of Merchant Taylors have made a grant of £10 10s. to the After-Care Association for Poor Persons Discharged Recovered from Asylums.

During the year 1899-1900 the number of patients who were admitted to the eleven Dublin hospitals under the jurisdiction of the Dublin Hospital Board was 12,644. Exclusive of incurables the mortality was 5·52 per cent.

The joint committee of the three Unions—West Derby, Toxteth, and Liverpool—have decided to purchase land at Heswall for the erection of a tuberculosis hospital. Mr. C. H. Lancaster has been appointed architect.

The annual report of the Swansea Hospital shows that during the year 892 patients were admitted to the institution. For the preceding year the total was 898. The number of outdoor patients treated was 4,392. The annual income had increased over the past few years by £243 odd. The house to house collections reached £418.

The cost of the new building, including site, architect's fees, and fittings, of the York Dispensary has up to the present time amounted to £5,798, which has been met out of special funds collected for the purpose, a legacy of £2,000, and by saving for the past five years on the part of the institution a sum of about £200 per annum. At the present time there is a debt of £315, and it is estimated that the balance due to the contractors will be £700.

Some interesting figures were adduced in a paper read before a meeting of the Bradford Joint Hospital Board. It appears that there are 250 places of worship in Bradford, producing an average annual collection of £4 each, whilst the workshops, numbering about 1,500, produce an average of £1 10s. each. Of the 250 places of worship only 130 made collections, and of the workshops 350 contributed. This made a total collection of £3,165 amongst a population of 300,000, or about 2½d. per head.

### The Student of Medicine.

### APPOINTMENTS.

W. E. Alderson, M.D., M.S.Durham, appointed Honorary Physician to the Newcastle-upon-Tyne Dispensary. A. Bissett, M.B., Ch.B., appointed Resident Physician to the Aberdeen Royal Infirmary. W. F. Croll, M.B., Ch.B., appointed Resident Surgeon to the Aberdeen Royal Infirmary. J. F. Hodgson, M.B., Ch.B.Vict., appointed Assistant House Surgeon to the Oldham Infirmary. J. J. Hopkins, L.R.C.P., L.R.C.S.I., appointed Certifying Factory Surgeon for the Nos. 1 and 2, Castlebar Dispensary Districts and the Electoral Division of Bellavady, in the Union of Castlebar. J. W. Leech, M.D., B.S., F.R.C.S.Edin., appointed Honorary Assistant Surgeon to the Royal Infirmary, Newcastle-upon-Tyne. G. C. Thomas, M.B., C.M., M.R.C.P.Edin., appointed Registrar to the Chelsea Hospital for Women, Fulham Road.

### THE NURSE'S REPORT BOOK.

Foolscap 4to., Black cover, 6d. net, post free; or 12 for 4s. 6d., post free.

Compiled by K. H. (Cert.)

H. J. GLAISHER, 57, Wigmore Street, Cavendish Square, W.

**NOW READY,**

A REPRINT OF

### A NURSING HANDBOOK

TOGETHER WITH S. MAW, SON, AND THOMPSON'S

**COMPLETE CATALOGUE OF NURSING REQUISITES.**

The above work is now republished at a cost of 1s. per copy. Messrs. S. M., S., and T. have been compelled to make this charge on account of the extreme popularity of the work and the great expense incurred in its production.

***Post Free on receipt of 1s.***

Nurses' Bags.

### S. MAW, SON, and THOMPSON,

7 to 12, ALDERSGATE STREET, LONDON, E.C.

